# Pediatric Acute Promyelocytic Leukemia: Epidemiology, Molecular Features, and Importance of GST-Theta 1 in Chemotherapy Response and Outcome

**DOI:** 10.3389/fonc.2021.642744

**Published:** 2021-03-19

**Authors:** Francianne G. Andrade, Suellen V. M. Feliciano, Ingrid Sardou-Cezar, Gisele D. Brisson, Filipe V. dos Santos-Bueno, Danielle T. Vianna, Luísa V. C. Marques, Eugênia Terra-Granado, Ilana Zalcberg, Marceli de O. Santos, Juliana T. Costa, Elda P. Noronha, Luiz C. S. Thuler, Joseph L. Wiemels, Maria S. Pombo-de-Oliveira, The Brazilian Collaborative Study Group of Acute Leukemia, Sarkis Renata Alves

**Affiliations:** ^1^Pediatric Hematology-Oncology Program, Research Center, Instituto Nacional de Câncer, Rio de Janeiro, Brazil; ^2^Laboratory of Molecular Biology, Bone Marrow Transplantation Center, Instituto Nacional de Câncer, Rio de Janeiro, Brazil; ^3^Surveillance and Prevention, Instituto Nacional de Cancer, Rio de Janeiro, Brazil; ^4^Department of Pediatric Hematology-Oncology, Hospital Martagão Gesteira, Salvador, Brazil; ^5^Clinical Research Department, Research Center, Instituto Nacional de Câncer, Rio de Janeiro, Brazil; ^6^Center for Genetic Epidemiology, Keck School of Medicine, University of Southern California, Los Angeles, CA, United States

**Keywords:** acute pediatric leukemia, childhood Incidence, prognosis, PML-RARA fusion gene, glutathione S-transferase

## Abstract

Previous studies have suggested a variation in the incidence of acute promyelocytic leukemia (APL) among the geographic regions with relatively higher percentages in the Latin American population. We aimed to explore the population burden of pediatric APL, gathering information from the population-based cancer registry (PBCR) and the diagnosis of APL obtained through incident cases from a hospital-based cohort. The homozygous deletion in glutathione S-transferases (GSTs) leads to a loss of enzyme detoxification activity, possibly affecting the treatment response. Mutations in the *RAS* pathway genes are also considered to be a key component of the disease both in the pathogenesis and in the outcomes. We have assessed mutations in a RAS–MAP kinase pathway (*FLT3, PTPN11*, and *K*-/*NRAS*) and *GST* variant predisposition risk in the outcome. Out of the 805 children and adolescents with acute myeloid leukemia (AML) who are registered in the PBCR, 35 (4.3%) were APL cases. The age-adjusted incidence rate (AAIR) was 0.03 per 100,000 person-years. One-hundred and sixty-three patients with APL were studied out of 931 AML cases (17.5%) from a hospital-based cohort. Mutations in *FLT3, KRAS*, and *NRAS* accounted for 52.1% of the cases. Patients with APL presented a 5-year probability of the overall survival (OS) of 67.3 ± 5.8%. A *GST-theta 1* (*GSTT1*) null genotype conferred adverse prognosis, with an estimated hazard ratio of 2.8, 95% confidence interval (CI) 1.2–6.9. We speculate that the GSTT1 polymorphism is associated with therapeutics and would allow better OS of patients with APL with a GSTT1 null genotype.

## Introduction

Acute promyelocytic leukemia (APL) is a relatively uniform subtype of acute myeloid leukemia (AML) characterized by the accumulation of abnormal promyelocytes in the bone marrow (BM). In about >95% of the APL cases, a chimeric fusion gene that drives the pathogenesis originates in the chromosome translocation 15q24 and *PML-RAR*α is a successful target for therapy using all-trans retinoic acid (ATRA) and arsenic trioxide (ATO) (1). Epidemiological studies reported the APL incidence rate in the adult population of 0.32 per 100,000 persons in the USA (2), and a high frequency in the Latin American, Southern European, and African childhood population (3). A wide variation in the relative frequency of pediatric APL among the worldwide population suggests that genetic predisposition and/or environmental exposures may influence susceptibility to breakage at the site involved in translocation and that ancestry and exposures *via* occupations or other factors should be studied for APL risk.

Inherited polymorphisms in the enzymes that activate or detoxify chemotherapeutic agents and the enzymes that protect cells from a reactive oxygen species generated by drugs may also influence the effectiveness of environmental exposures on health, treatment efficacy, and long-term outcomes. Glutathione S-transferases (GSTs) are a family of phase II detoxification enzymes, which act either by the direct conjugation of a drug with glutathione or by neutralizing drug-induced reactive compounds. The homozygous deletion of GSTs (named as null genotype) leads to a complete loss of enzyme activity, possibly affecting the treatment response (4). A profound polymorphic variance of these enzymes may be significant within promyelocytes, which are metabolically active cells containing high myeloperoxidase levels necessary for their primary functions, within primary azurophilic granules (5). In the context of somatic aberrations, APL is almost universally defined by the *PML–RAR*α fusion gene, while the RAS pathway mutations are additionally a key component of the disease both in the pathogenesis and outcome. Mutations in the RAS–MAP kinase pathway genes are recurrent in leukemia, acting in leukemogenesis and the outcome (6).

To strengthen the ties of observational and molecular epidemiological studies of childhood APL in the Latin population, we explored the population burden of pediatric APL in Brazil, gathering the information from a population-based cancer registry (PBCR) and the APL diagnosis obtained through the incident cases from a hospital-based cohort. We have assessed both the mutations in the RAS–MAP kinase pathway (*FLT3, PTPN11*, and *K-/NRAS*) and the predisposition risk of a GST variant in the outcome. We built this data with our unique and complementary network study group of childhood leukemia in Brazil to assess the impact of biomarkers in the APL cases in the overall survival (OS) (7).

## Results

### Acute Promyelocytic Leukemia Incidence Rate

**Figure 1:**
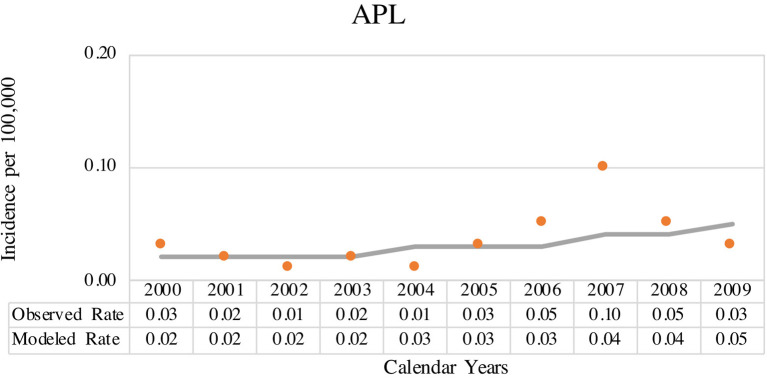
Joinpoint analysis for age-adjusted incidence rate (AAIR) (0–19 years, standard world population) from acute promyelocytic leukemia (APL) in Brazilian population-based cancer registry (PBCR), 2000–2009. Solid circles indicate the data for observed age-adjusted rate and solid line indicates the data for modeled age-adjusted rate.

The coverage area of PBCR is estimated to be about 20% of the Brazilian population. Table 1 shows the number of APL cases, sex ratio, age-specific incidence rates (ASIRs), crude rates, and age-adjusted incidence rates (AAIRs) for the Brazilian regions. Out of 805 AML cases in children and adolescents who were registered in the PBCR (2000–2009), 35 cases were APL (4.3%) (data not shown). About <3% of the patients were identified with the death certificate. The male:female ratio was 1.2:1.0. The AAIR of APL was 0.03 per 100,000 person-years [95% confidence interval (CI) 0.03–0.03]. AAIR varied according to the macro-geographical region, with a higher incidence in the South region of Brazil in comparison with other areas. The incidence trends in AAIR (0–19 years) for the APL cases in the whole country remain stable throughout the period. The annual percent change (APC) is 14.1% (95% CI −2.9–34.0), although we observed fluctuations in all the Brazilian regions (Figure 1; Supplementary Table 1).

**Table 1:** Data on the incidence rate of APL cases from 15 Brazilian population-based cancer registries, 2000–2009.

					**ASIR**				**Crude**	AAIR^**^
**Region**	Coverage^*^ **(%)**	**Total,** **n (%)**	**Male,** **n (%)**	**Female,** **n (%)**	**0–4 years**	**5–9 years**	**10–19 years**	**0–19 years**	**0–19 years**	**95% CI**
North	28	3 (8.6)	1 (33.3)	2 (66.7)	0.00	0.00	0.04	0.02	0.02	0.02**–**0.02
Northeast	13	4 (11.4)	4 (100.0)	0 (00.0)	0.03	0.05	0.01	0.02	0.02	0.02**–**0.02
Center	21	2 (5.7)	2 (100.0)	0 (00.0)	0.00	0.06	0.03	0.03	0.03	0.03**–**0.03
Southeast	23	11(31.4)	5 (45.5)	6 (54.5)	0.01	0.02	0.03	0.02	0.02	0.02**–**0.02
South	13	15 (42.9)	7 (46.7)	8 (53.3)	0.00	0.16	0.21	0.15	0.13	0.13**–**0.14
Brazil	20	35 (100.0)	19 (54.3)	16 (45.7)	0.01	0.04	0.04	0.03	0.03	0.03**–**0.03

* Percentage of the population covered;

** World standard population. Registries by region of Brazil: North (Belém, include Ananindeua city; and Manaus); Northeast (Aracaju; Fortaleza; João Pessoa; and Recife); Center-West (Cuiabá, include Várzea Grande city; and Goiânia); Southeast (DRS Barretos, include Altair, Bebedouro, Cajobi, Colina, Colômbia, Guaíra, Guaraci, Jaborandi, Monte Azul Paulista, Olímpia, Severínia, Taiaçu, Taiúva, Taquaral, Terra Roxa, Viradouro, and Vista Alegre do Alto cities; Belo Horizonte; Vitória, include Cariacica, Vila Velha, Fundão, Guarapari, Serra, and Viana cities; Jahu; and São Paulo); South (Curitiba and Porto Alegre).

*N, total number cases; APL, acute promyelocytic leukemia; AAIR, age-adjusted incidence rates; ASIRs, age-specific incidence rates; CI, confidence interval*.

### Acute Promyelocytic Leukemia in a Hospital-Based Cohort

We have characterized 163 APL (17.5%) of 931 AML cases in a time frame of 17 years in the macro-geographical Brazilian regions, as shown in Table 2. Patients presented a median age at the diagnosis of 10.1 (0.75–19) years. The male:female ratio was 1.2:1.0 with a prevalence of non-blacks (89.4%). The prevalence of APL cases from the Northeast and Southeast regions was 41.1 and 28.2%, respectively; the South region presented the lower frequency (2.5%). Cases were sent from 33 medical centers, including seven hospitals that fed the PBCRs information data. We observed an increased frequency of APL cases from 2010 to 2018.

**Table 2:** The distribution frequency of APL diagnosed according to microregion, age, sex, and ethnicities, Brazil, 2002–2018.

**Region**		**Centers of treatment,** **n (%)**	**Total,** **n (%)**	**Male, n (%)**	**Female,** **n (%)**	**0–4 years** **n (%)**	**5–9 years** **n (%)**	**10–19 years** **n (%)**	**Blacks** ** n (%)**	**Non-blacks,** **n (%)**
North										
	2002–2009	0 (0.0)	0 (0.0)	0 (0.0)	0 (0.0)	0 (0.0)	0 (0.0)	0 (0.0)	0 (0.0)	0 (0.0)
	2010–2018	3 (100.0)	3 (100.0)	3 (100.0)	0 (0.0)	2 (100.0)	0 (0.0)	1 (100.0)	0 (0.0)	3 (100.0)
Northeast										
	2002**–**2009	9 (47.4)	31 (46.3)	19 (46.3)	12 (46.2)	8 (50.0)	10 (45.5)	13 (44.8)	7 (70.0)	19 (36.5)
	2010**–**2018	10 (52.6)	36 (53.7)	22 (53.7)	14 (53.8)	8 (50.0	12 (54.5)	16 (55.2)	3 (30.0)	33 (63.5)
Center-west										
	2002**–**2009	3 (42.9)	11 (25.6)	7 (33.3)	4 (18.2)	0 (0.0)	3 (27.3)	8 (27.6)	1 (50.0)	8 (21.1)
	2010**–**2018	4 (57.1)	32 (74.4)	14 (66.7)	18 (81.8)	3 (100.0)	8 (72.7)	21 (72.4)	1 (50.0)	30 (78.9)
Southeast										
	2002**–**2009	8 (44.4)	16 (34.8)	6 (26.1)	10 (43.5)	1 (9.1)	8 (66.7)	7 (30.4)	1 (33.3)	12 (30.0)
	2010**–**2018	10 (55.6)	30 (65.2)	17 (73.9)	13 (56.5)	10 (90.9)	4 (33.3)	16 (69.6)	2 (66.7)	28 (70.0)
South										
	2002**–**2009	1 (33.3)	1 (25.0)	1 (50.0)	0 (0.0)	0 (0.0)	1 (50.0)	0 (0.0)	0 (0.0)	0 (0.0)
	2010**–**2018	2 (66.7)	3 (75.0)	1 (50.0)	2 (100.0)	0 (0.0)	1 (50.0)	2 (100.0)	1 (100.0)	2 (100.0)
Brazil		33 (100.0)	163 (100.0)	90 (55.2)	73 (44.8)	32 (19.6)	47 (28.8)	84 (51.6)	16 (10.6)	135 (89.4)

The APL cases were clinical-morphological characterized by hyper granular subtype (86.5%) median of white blood cells (WBCs) count of 13.1 × 10^9^/L (min,1 × 10^9^/L, max 800 × 10^9^/L). Twenty-two patients died in the first 10 days after the clinical diagnosis. Early death occurrence was observed in 10 patients who died before starting any treatment. The main cause of death was hemorrhage (60.8%) next to septicemia complications (29.4%). About 130 patients (130/151, 86.1%) presented the *PML–RAR*α fusion, with breaks distributed in intron 3 [breakpoint cluster regions (bcr) 3] of the PML gene (30/53, 56.6%), intron 6 (bcr 1; 18/53, 34.0%), and exon 6 (bcr 2; 5/53, 9.4%). Overall, the mutations in *FLT3, KRAS*, and *NRAS* accounted for 52.1% of the cases.

#### FLT3 Mutations in Pediatric Acute Promyelocytic Leukemia

As shown in Table 3, 52 patients (52/120, 43.3%) presented *FLT3* mutations, including 6 (5.0%) mutations in the tyrosine kinase domain (TKD) in codon 835 and 46 (38.0%) internal tandem duplications (ITDs) in exons 11/12. Patients with *FLT3* mutations had a median age at the diagnosis of 11.2 years, similar to the observation done for the patients with *FLT3* wild-type (9.8 years; *p* = 0.264); cases at an early age (≤ 2 years of age) presented no mutations. Most patients with *FLT3* mutations presented high WBC count (>10 × 10^9^/L) and low platelet count (≤ 40 × 10^9^/L) (Table 3; Supplementary Figure 1). We found an association of *FLT3* mutations with a variant promyelocytic subtype (M3v or microgranular) in 21.2% of the cases and a high frequency of *PML-RAR*α bcr 3; most patients without the mutations presented *PML-RAR*α bcr 1 (Table 3). The median of the allelic frequency ratio of *FLT3* ITD mutations was 0.35. We used the median as a cutoff to compare low (≤ 0.35) and high (>0.35) allelic ratios according to the demographic and laboratory features. As described in Supplementary Table 2, we observed an association of high allelic ratio of *FLT3* ITD and patients who were aged from 2 to 10 years old, males, as well as having a high WBC count (>10 × 10^9^/L).

**Table 3:** Characteristics of APL according to FLT3 mutations (ITD/DTK).

	Total^*^, n (%)	***FLT3*^**mut**^, n (%)**	***FLT3*^**wt**^, n (%)**	**p**
Median age at diagnosis (years)	10.1	11.2	9.5	0.197
Age strata (years)				0.087
≤ 2	8 (4.9)	0 (0.0)	5 (7.4)	
>2–10	71 (43.6)	21 (40.4)	31 (45.6)	
≥11	84 (51.5)	31 (59.6)	32 (47.1)	
Sex				0.292
Females	73 (44.8)	18 (34.6)	30 (44.1)	
Males	90 (55.2)	34 (65.4)	38 (55.9)	
Race				1.000
Blacks	16 (10.6)	5 (10.4)	6 (9.7)	
Non-Blacks	135 (89.4)	43 (89.6)	56 (90.3)	
Median WBC count (× 10^9^/L)	13	31	7.1	0.002
WBC count at diagnosis (× 10^9^/L)				0.001
≤ 10	74 (46.8)	14 (27.5)	39 (59.1)	
>10	84 (53.2)	37 (72.6)	27 (40.9)	
Platelet, median (× 10^9^/L)	22,500	21,000	30,500	0.118
Platelet (× 10^9^/L)				0.011
≤ 40	105 (70.0)	39 (83.0)	40 (60.6)	
>40	45 (30.0)	8 (17.0)	26 (39.4)	
Morphologic subtype				0.028
Hypergranular	141 (86.5)	41 (78.8)	63 (92.6)	
Microgranular	22 (13.5)	11 (21.2)	5 (7.4)	
*PML* breakpoint region				<0.0001
Bcr 1	18 (34.0)	2 (9.5)	13 (59.1)	
Bcr 2	2 (9.4)	0 (0.0)	4 (18.2)	
Bcr 3	3 (30.0)	19 (90.5)	5 (22.7)	
*RAS* MUTATIONS				0.702
Mutated	11 (9.1)	2 (5.1)	5 (8.2)	
Wild-type	110 (90.9)	37 (94.9)	56 (91.8)	
*GSTT1* polymorphism				0.556
Non-null	77 (67.0)	25 (64.1)	35 (70.0)	
Null	38 (33.0)	14 (35.9)	15 (30.0)	
*GSTM1* polymorphism				0.969
Non-null	66 (57.4)	22 (56.4)	28 (56.0)	
Null	49 (42.6)	17 (43.6)	22 (44.0)	
Early death^**^				0.003
Yes	24 (17.6)	22 (47.8)	12 (20.7)	
No	112 (82.4)	24 (52.2)	46 (79.3)	
Total	163 (100)	52/120 (43.0)	69120 (57.0)	

* We investigated 120 cases for FLT3 mutations.

** *Death in the first 10 days after diagnosis. Survival data were available from 136 patients. Mut, mutated (internal tandem duplications (ITDs) and tyrosine kinase domain (TKD) in codon 835); n, number of cases; WBC, white blood cell; wt, wild-type. All p-values were calculated using valid data and considered to be significant if < 0.05*.

#### Genetic Susceptibility

A *GSTM1* null genotype occurred in 42.6% (49/115) of the cases and 40.3% (160/397) of the controls (*p* = 0.658) while a *GST-theta 1* (*GSTT1*) null genotype was present in 33.0% (38/115) of the cases and 22.7% (90/397) of the controls (*p* = 0.024). We estimated the risk associations between genetic polymorphisms and APL as odds ratio (OR) 1.10 (95% CI 0.72–1.68) for the *GSTM1* null genotype and OR 1.68 (95% CI 1.07–2.65) for the *GSTT1* null genotype, respectively (Supplementary Table 3). We have tested risk associations between genetic alterations in *GSTM1*/*GSTT1* and patients with APL harboring *FLT3* mutations. There was no association between *GSTM1*/*GSTT1* deletions and *FLT3* mutations (crude OR = 0.98, 95% CI 0.42–2.29 and 1.31, 95% CI 0.54–3.19, respectively).

### Survival

**Figure 2:**
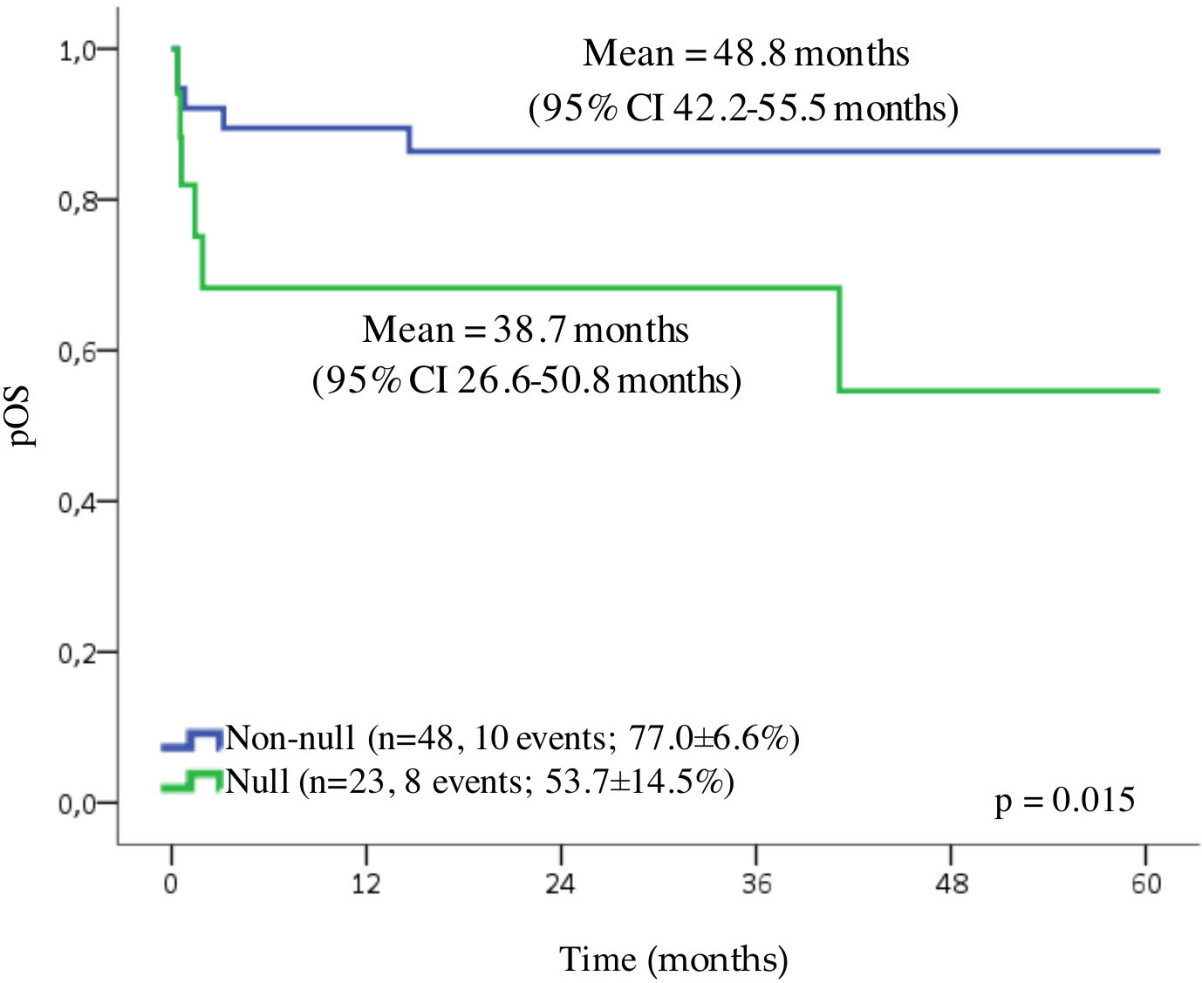
Overall survival (OS) of pediatric cases of APL. Probability of the OS, according to *GSTT1* polymorphisms.

The data from 136 patients (83.4%) were available for the survival analysis. In the first 10 days after the diagnosis (early death), death affected 17.6% (24/136) of the patients, and we excluded these cases from the analysis. Patients with APL presented the probability of OS for 5 years of 67.3 ± 5.8% and a mean of 45.3 months (95% CI 40.2–50.4 months). Because of a small number of cases in each Brazilian geographic region category, we grouped patients in three localities (North/Northeast, Center-West, and South/Southeast). We observed no statistically significant differences in the outcome according to the locality of treatment, as well as to age, WBC and platelet counts, and the presence of *RAS* or *FLT3* mutations. Although the patients with an undetected *PML-RAR*α presented a slightly lower probability of OS, the difference was not statistically significant (Supplementary Table 4). A *GSTT1* null genotype conferred adverse prognosis compared to wild-type genotype (probability of OS for 5 years 48.2 ± 13.4% and 80.5 ± 6.1%, respectively; *p* = 0.015) shown in Figure 2. The Cox regression model estimated the crude hazard ratio for *GSTT1* deletions of 2.8, 95% CI 1.2–6.9, *p* = 0.021. No adjusting variable was retained in a multivariate model (Supplementary Table 5).

## Discussion

Here, we report a comprehensive overview of the diagnostic characteristics and survival of APL pediatric cases in Brazil for the first time. Using two large data sources, we described the incidence of APL in children and adolescents from a population with Latino genetic background. We tested the hypothesis that polymorphisms in the genes of metabolic enzymes, i.e., GST, might affect the detoxification of chemotherapeutic compounds and found that a complete loss of the *GSTT1* is associated with poor outcome.

Acute promyelocytic leukemia is a rare disease with an incidence rate in the US population of 0.27 per 100,000 in 2000–2008, increasing over time (0.11 per 100,000 person-years in 1975–1990). In young patients (<20 years of age), the disease is far rarer, with 0.06 per 100,000 compared to 0.36 per 100,000 among the patients diagnosed at ≥60 years of age, respectively (2). From PBCR with a coverage of 20% of the Brazilian population, we found an AAIR among the children of 0.03 per 100,000 persons in 2000–2009. An incidence of childhood cancer has been previously reported for Brazil; however, this is the first time APL was stratified from AMLs and compared to a robust hospital-based population (8–10). Other population-based studies have reported APL age-adjusted incidence of 0.08 cases per 100,000 person-years in Canada (11) and, in children, 2.5 per million person-years in Italy (12), 1.39 per million people in Spain, 0.54 per million person-years in Great Britain, 0.4 per million person-years in France, and an overall rate of 0.0–0.1 per 100,000 in the European population (13, 14). Environmental exposures and genetic predisposition, which make chromosomic sites to involve in the *PML-RAR*α origin more prone to break in specific settings, may explain the epidemiological variation giving a hint into the pathogenesis of APL (15). As previously reported, region-specific environmental exposures might be associated with the underlying causal factors. The high rate in the South of the country might result from the main local economic activities such as agriculture and oil and gas industries (8). We observed a contrast in the incidence rate in the South region of Brazil in comparison to the other areas. Historically, the ancestry in the North has a considerable influence on Amerindian roots, the Northeast presents a strong African influence from slavery, and the European immigrants mostly settled in the South. European ancestry proportions range from 60.6% in the Northeast to 77.7% in the South (16). The macro-regions are primarily for statistical purposes and intriguingly mirror environmental exposures, and genetic background, which might result in the high incidence of APL observed in the South. A population-based study in Brazil has previously demonstrated regional differences in the incidence of AML (including APL) (9). The bias might be in the better quality of PBCR from the South than the other regions, measured by the percentage of the cases confirmed microscopically and <20% of the cases collected through death certificates. As noted by Feliciano et al. (8), the rates of unspecified leukemia were inversely proportional to the APL rates in the different Brazilian geographic regions, which led to the conjecture that the cases of APL could be included in the group of unspecified leukemia. However, we cannot rule out some case capture differences in the different regions of Brazil included in this study as another potential explanation of varied rates among the areas.

With an awareness of the potential challenges of the PBCR data representation of the Brazilian population, we also analyzed a hospital-based cancer registry covering >90% of pediatric leukemia in Brazil. PBCR mostly covers the people living in capital cities, and the hospital-based cancer registry does not exclude the patients according to the place of residence. We have built up a large population of leukemia patients over the past two decades by using an innovative clinical path resource for pediatric oncologists in Brazil, enhancing clinical treatments, and providing patient participation opportunities in epidemiologic studies (7). Our diagnostic resource offers a quality support mechanism and may yield a complete case capture. The rarity of an APL diagnosis directed our incidence estimates by calculating the proportion of APL among the AML cases. In combination with the immunophenotypic and molecular characterization, the well-defined morphological features of blast cells have reliably allowed the identification of APL.

We might explain the differences in the absolute number of APL cases between PBCR and a hospital-based cancer registry by using the different coverages of the registries and the period of analysis, and the inherent classification problems in the data sources. Despite the overlapping cases from the hospital based and the PBCR, several PBCR still lack this criteria and APL cases might be included in the AML group and the “no otherwise specified” entity (ICD-O-3 9861) (17–19).

We reported a proportion of 17.5% of APL among the AML cases referred to 52 Brazilian institutions. Jacomo et al. (20) have reported that the patients with APL represented 28.2% of the AML cases from 12 Brazilian institutions. This information could be biased by using the different patient referral patterns to specific hospitals and by including only the patients who were 15 years or older (15). The highest proportions (15–58%) of childhood APL were reported in the South and Central America studies, including Costa Rica, Chile, Bolivia, Guatemala, Honduras, and El Salvador. In African countries, APL represented around 15–20% of pediatric AML cases (Nigeria, South Africa, Tunisia, and Sudan), 10–15% of the AML cases in Egypt, and 5–10% of the AML cases in Malawi. Proportions of APL were similarly variable in the Middle East (3.4% in Saudi Arabia and 34.5% in Iraq) and Asia where China and Pakistan describe a proportion of 36.2 and 43.5%, respectively (3).

The translocation (14, 16) (q24;q21) that results in the chimeric gene/protein *PML-RAR*α is a defining abnormality of APL. Using the techniques of fluorescence *in situ* hybridization (FISH) and reverse transcriptase PCR (RT-PCR), we could not detect *PML-RAR*α in 13.9% of the cases. Zhao et al. (21) described the absence of *PML-RAR*α in 18.8% of pediatric APL cases and the FISH and RT-PCR sensitivities as 91.30 and 88.64%, respectively. In the conflicting results, a next-generation sequencing performed in seven FISH/RT-PCR *PML-RAR*α negative samples revealed six *PML-RAR*α samples and one *NPM-RAR*α (21). Atypical *PML-RAR*α results in negative RT-PCR and/or FISH are a consequence of complex rearrangements, alternative splicing within *PML* or *RAR*α, as well as through insertions/deletions beyond the limit of resolution (22–25). The effects of storage temperature and duration of whole blood on RNA quality might also affect the assay results, especially in a multicentric study in which the samples undergo environmental variations (26). When a next-generation sequencing is not available, clues to the diagnosis are the highly characteristic morphological, immunophenotypic, and clinical features (27).

The breakpoint in the *RARa* gene falls into intron 2 while the breakpoints in *PML* are distributed in intron 6 (bcr 1), exon 6 (bcr 2), and intron 3 (bcr 3), in 55, 8, and 40% of cases, respectively (28). We observed frequencies of bcr 1 in 34.0%, bcr 2 in 9.4%, and bcr 3 in 56.6% of the cases. Previous studies reported bcr 1 in 42.9–75% of the patients, with the highest proportions in Hispanic/Latino patients (15, 29–33). Our concordant findings fall into *FLT3* wild-type patients (bcr 1 = 59.1%), meaning that secondary mutational events might give a growth advantage for pre-leukemic clones rather than a genetic background of a specific population. The bcr clusters of *PML* and *RARa* in therapy-related APL in genomic regions that are particularly susceptible to cleavage by using topoisomerase II inhibitors corroborates this hypothesis (3).

While all patients with APL will have a private or low frequency of recurrent mutations, we expect all APLs to harbor the mutations in a limited number of pathways, which reflect the homogeneous nature of the disease. Our findings of recurrent mutations in *KRAS/NRAS* genes in 8.8% of the APL cases were slightly lower than the observation by others, and the mutations in *FLT3* (43.3%) were similar to the previous studies (20–53%) (21, 33–35). A higher frequency of *FLT3* mutations in older patients is believed to result from a multistep process of the accumulation of secondary mutations (36). Other authors also reported the association of *FLT3* mutations with high WBC count, bcr 3 in *PML* gene, and microgranular morphology (29, 37–39). For *FLT3* ITD mutations, the allelic ratio refers to the number of ITD-mutated alleles compared to the wild-type allele number. This ratio is not only influenced by the amount of malignant vs. non-malignant cells in the tested sample but also by the percentage of cells with 0, 1, or 2 mutated alleles. The allelic ratio reflects the clonal burden of the *FLT3* ITD-mutated cells within the leukemia cell population (40). Like secondary events, *FLT3* mutations occur in a subset of the leukemic cell population only, resulting in both the upregulation of genes associated with cell division and proliferative signals *in vitro*. Thus, *FLT3* mutations could readily account for the *in vivo* association with higher WBC count and are at very high risk for death (38, 41). On the contrary to the previously described ones, we did not find an association of *FLT3* mutations with the OS (33).

Despite laboratory and molecular support for the diagnosis, our early death rate was higher compared to clinical trials. Rego et al. (42) described that 15% of the deaths occurred between a diagnosis and the first morphologic evaluation of BM (30 days), with 5% (9 cases) of the deaths within the first 7 days of treatment from the International Consortium on APL gathering patients >15 years of age from Brazil, Mexico, Chile, and Uruguay. Adès et al. (43) reported 2.8 and 6.8% of the deaths in the first 30 days of treatment in the LPA 99 trial (Spain, Argentina, The Netherlands, and the Czech Republic) and APL 2000 trial (France, Switzerland, and Belgium), respectively. Avvisati et al. (44) reported 5.5% (44 patients) of the deaths during the induction, and three patients died before starting the treatment in the AIDA 0493 trial (Italy, The Netherlands, and Germany). The causes of early death include a delay in referral to specialized centers and in recognizing APL due to its rarity, the availability of ATRA in the centers, and supportive care management. Access to such supportive care is more complicated outside the major cities in Brazil. The international effort to recognize and treat APL reduced early mortality and increased OS in developing countries. Action has been made to ensure prompt availability of ATRA in all centers, the definition of pre-existing transfusion medicine services, and hematology laboratories able to perform basic fluorescence microscopy, data report, and the establishment of a reference laboratory for molecular diagnosis (42). Further educational training of healthcare providers to recognize APL as a medical emergency will impact early death and improve survival rates mainly because most of the patients are first admitted in emergency rooms or intensive care units (45).

In the present study, OS was lower compared to collaborative studies and trials from developed countries in which a 5-year OS for APL reaches ~95% (46, 47). Our patients treated according to the BFM-based protocol received more intensive treatment than those treated according to the GIMEMA-based protocol. Nowadays, both GIMEMA and BFM study groups recommend low or no dose chemotherapy for low-risk patients and ATRA combined with ATO for APL treatment (48, 49). In Brazil, physicians use ATO as a first-line therapy for the last 3 years, and a uniform protocol has been developed in Latin America for pediatric APL (50).

After overcoming the complications in the first month of treatment, some patients will face failure in the treatment and risk relapse attributable to genetic susceptibility. Davies et al. (51) showed for the first time an association of a *GSTT1* null genotype and an increase of deaths in remission patients with AML after intensive chemotherapy, including a high-dose anthracycline and cytarabine. GSTs are phase II detoxification enzymes that metabolize cancer chemotherapeutic agents, insecticides, herbicides, carcinogens, and oxidative stress products, catalyzing the conjugation of glutathione to these substances (4). Anthracyclines used in treating leukemia, such as daunorubicin, generate high levels of reactive oxygen species and activate GSTs activity, which protects cells (52). Polymorphic gene deletions in *GSTT1* or *GSTM1* result in the absence of protein and may thus play a role in both leukemogenesis and in response to the treatment (53, 54). We found evidence of an association here between the *GSTT1* polymorphisms and the outcome of APL. However, current and previous studies are still somewhat preliminary to prove an association of *GSTT1* status and the outcome of acute leukemia due to small sample sizes, the differences in the chemotherapy regimens, and the age of the population included in the analyses (54–56). The strengths of the study are the clinical and laboratory characterization of a large cohort of children with APL with the relevance of GST polymorphisms associated with the outcome. The original results support partially our hypothesis. We described here an overview of the epidemiological, molecular, and clinical features of pediatric APL in Brazil. We confirmed a high proportion of APL among the AML cases in the Brazilian population, suggesting the influence of genetic background in leukemogenesis although the multicenter and retrospective data collection limits our work. This approach might be affected by slight differences in treatment strategies, making us unable to evaluate the therapeutic protocol efficacy and causes of early death. However, we found new evidence of chemotherapy metabolism effects on survival. A *GSTT1* null genotype, which results in a loss of enzyme activity, was associated with a poorer response to therapy. Since only the individuals who had a homozygous-variant genotype (i.e., completely deficient) were classified and the heterozygous individuals were not distinguished from the wild-type patients, we believe there is an underestimation of the impact of *GSTT1* on the outcome. A limitation of this study should be addressed to the results obtained in the PBCR inconsistencies which are evident when compared with the data obtained in hospital-based data (HBD) and with other PBCR worldwide. The differences between the PBCR and the HBD result from a variation in the reported diagnosis, for instance, in the PBCR the majority of APL either reported in AML or in not-otherwise specified categories, while in the HBD the diagnosis is based on cytogenetic-molecular tests.

## Materials and Methods

### Study Subjects

**Figure 3:**
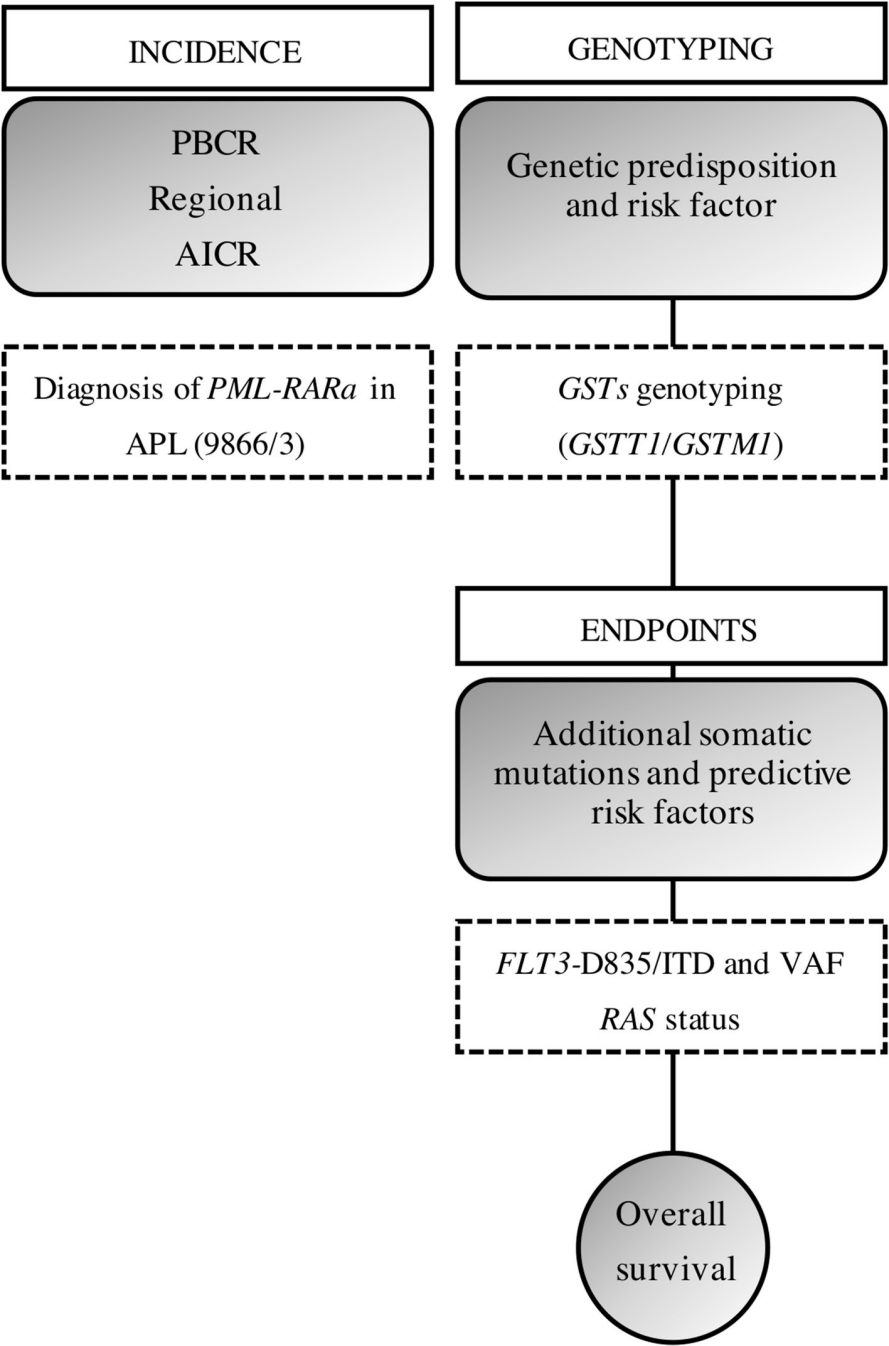
Study design. We gathered the information from a PBCR and the diagnosis of obtained through the incident cases from a hospital-based cohort. AAIR, age-adjusted incidence rate; ASIR, age-specific incident rate; ITD, internal tandem duplications; VAF, variant allelic frequency.

Our approach was to bring together the data on PBCR, APL diagnosis, biomarkers characterization, and genetic predisposition. Figure 3 shows a brief study design and how the data were built.

#### Population-Based Cancer Registries

The incidence data of APL (patients aged 0–19 years at the diagnosis) were obtained from databases of 15 PBCR across the Brazilian region from the period of 2000–2009 (8). Brazilian cancer registries correspond to local records by State, located in Capital and metropolitan surroundings, which gather the information on incidence cancers in the contributing registration areas. The PBCR distribution represents the Brazilian macro-geographical regions (shown in Supplementary Figure 2). The data were collected according to the International Agency for Research on Cancer (IARC) requirements for Cancer Registries according to quality data parameters, such as percentage of the AML cases diagnosed and confirmed microscopically, and <20% of those identified from the death certificate only (57, 58). Cases were classified according to the Third Edition of the International Classification of Diseases for Oncology (ICD-O-3/9866). Only data sets with high-quality data were eligible for inclusion in the analyses. Leukemia data were extracted from the *Instituto National de Câncer* and population demography from the *Instituto Brasileiro de Geografia e Estat*í*stica* website (https://www.inca.gov.br/BasePopIncidencias/Home.action; https://datasus.saude.gov.br).

#### Hospital-Based Cohort

Acute promyelocytic leukemia in children and adolescents following the PBCR age strata was diagnosed (2002–2018) in 33 Brazilian Oncological Centers and in the Pediatric Hematology-Oncology Program, Instituto Nacional de Câncer, Brazil, that is a reference laboratory for molecular diagnosis and pathology of childhood leukemia (59). Inclusion criteria were the cases with a diagnosis of APL according to morphology, immunophenotyping, or the presence of *PML-RAR*α in BM and peripheral blood (PB) samples from the patients aged 0–19 years old. Molecular analysis for the detection of *PML-RAR*α was based on the results of the good quality of a diagnostic material. The data set with demographic and clinical information were obtained from the clinicians at the time of diagnosis and complementary data during the follow-up of patients, such as the occurrence of relapse (if any, with data and local) and the status of the patient (death/alive).

Biological specimens (BM and PB samples) were collected previously for any chemotherapy treatment and to test minimal residual diseases. A predefined set of variables was collected, including sex, age, ethnicity, place of residence, significant clinical symptoms, WBC count, platelets count, and blast cells percentage. The institution staff of the patients provided these data *via* an online system (https://imunomolecular.inca.gov.br/).

The diagnostic algorithm tests included BM morphology, immunophenotyping, and molecular cytogenetics as described in Supplementary Figure 3. Briefly, samples were sent with the clinical report and suspicious of APL. Samples were immediately prepared for the identification of a *PML–RAR***α** fusion gene by using FISH. Immunophenotyping was performed and the APL diagnosis was released to clinicians in about12 h after the samples were received.

### Molecular Diagnosis

Total RNA was purified from BM cells using TRIzol™ reagent (Invitrogen, Carlsbad, CA, USA) and directed for a complementary DNA (cDNA) synthesis following a transcriptase reverse enzyme addition. cDNA integrity was evaluated throughout the GAPDH amplification. Genomic DNA was purified from the BM by using a TRIzol™ reagent (Invitrogen, CA, USA), the QIAmp DNA Blood Mini Kit (Qiagen, CA, USA), or the HiYield Genomic DNA Mini Kit (Real Biotech Corporation, Taiwan).

*PML-RAR*α was identified by using FISH with commercial probes (PML/RARa Translocation Probe Dual Fusion; Cytocell, Cambridge, UK). A RT-PCR was performed for the identification of *PML-RAR*α according to a previous study (28). The bcr is localized within a 15 kb of the *RAR*α intron 2 and three regions of the *PML* locus in intron 6, exon 6, and intron 3, respectively, named as bcr1, bcr2, and bcr3. A combination of four primer pairs was used to amplify RT-PCR products with different sizes allowing to identify transcripts with breaks in intron 6, exon 6, and intron 3 of *PML* and the variable breakpoint positions. Primer sequences were described by Van Dongen et al. (28). A nested amplification was included to improve the sensitivity of the reaction. Both reactions followed RT-PCR temperatures and cycle times: initial melting: 95°C for 30 s, 35 cycles of 94°C for 30 s (melting), 65°C for 60 s (annealing), and 72°C for 60 s (extension). The evaluation of the PCR products was performed with 10 ml of the products in a 1.5% agarose gel electrophoresis. The *PML-RAR*α screening has been included in the APL diagnosis algorithm since 2009. The used positive control was the sample from APL with *PML-RAR*α positive using FISH methods and confirmed by direct sanger sequencing.

### Mutation Detection

The mutations in hotspot regions of RAS–MAP kinase pathway signaling genes (*FLT3, NRAS, KRAS*, and *PTPN11*) were analyzed by direct sequencing according to a previous study (59). The analysis included the cases with available biological materials (valid data; Supplementary Table 6).

Briefly, *FLT3* mutations were examined at the TKD in codon 835 and the juxtamembrane domain in exons 11/12 as ITD. The variant allele frequency ratio of *FLT3* ITD was assessed by a 6-carboxyfluorescein and PCR forward primer 5-end-labeled fragment analysis. About 1 μl of the product was analyzed in ABI3130XL (Applied Biosystems, CA, USA). The area of mutant peaks on the electropherogram was analyzed using the ChimerMarker® software (SoftGenetics, PA, USA). The *FLT3* ITD mutant allelic burden was calculated as the ratio of the area under the curve of mutant and wild-type alleles (mutant/total). *NRAS*/*KRAS* status was determined by searching the mutations in exon 1 (codons 12/13), and *PTPN11* mutations were screened in exons 3 and 13 (60). Primers and PCR conditions are described in Supplementary Table 7.

### Glutathione S-transferase Genotyping

*GSTM1* and *GSTT1* homozygous deletions were detected by using a multiplex PCR as described in a study (61). The null genotype definition consisted of both copies of the genes *GSTM1* or *GSTT1* to be deleted while the non-null genotype was defined as at least one copy of the gene presented. The attributable risk conferred by genetic susceptibility was tested in a case-control study. An unmatched control group of healthy samples was used to estimate the susceptibility risk. It consisted of 416 umbilical cord blood samples randomly selected from the National Umbilical Cord and Placental Blood Bank. The samples were collected from healthy newborns at birth and are described in a study (62).

### Treatment

Patients were not enrolled in a supervised clinical trial. APL management initially followed the same recommendations in the International Consortium on APL study of adult patients (50). Overall, the pediatric Brazilian institutions have established therapeutic protocols guided by two international clinical protocols: (i) the GIMEMA-based protocol that consisted of induction phase with ATRA and anthracycline, followed by consolidation phase with ATRA/Anthracycline and Cytarabine (three cycles), and maintenance with ATRA/Mercaptopurine and methotrexate (2 years) (48) and (ii) the BFM-AML-based protocol that consisted of the management of the patients with APL as low-risk AMLs with the addition of ATRA. Briefly, the induction-phase included ATRA, anthracycline, cytarabine, etoposide, followed by consolidation with ATRA, alternating anthracycline, cytarabine, and etoposide (three cycles), and 1-year maintenance of continuous thioguanine and one low dose of cytarabine monthly (49, 50).

### Ethical Research

All collaborators have reviewed the ethical board and approved the APL treatment care according to the local laws and guidelines, following the Declaration of Helsinki. Informed consent was obtained from the parents of the children for the research assessments. The Ethical and Research Committee approved this study of the *Instituto National de Câncer* (#186.688).

### Statistical Analysis

#### Population-Based Cancer Registries

Incidence rates were calculated as the annual number of cases per 100,000 persons per year, according to the geographical regions (North, Northeast, Center-West, Southeast, and South), based on a 10-year period of available information for all PBCR (2000–2009). AAIR per 100,000 person-year was calculated using the World Standard Population as described in a previous study (8). The 95% CI AAIR was calculated using the Poisson approximation. Incidence trends were performed using the Joinpoint regression model to identify the changes in APL incidence rates over time. To minimize the variability inherent in a small number of observations, the logarithm of the rates was used in the total data set. APC and average annual percent ranges (AAPC) were estimated and reported with corresponding 95% CI for each of the linear segments identified between two Joinpoints. The software was provided by the Surveillance Research Program of the US National Cancer Institute, version 4.6.0.0 (https://surveillance.cancer.gov/joinpoint).

#### Hospital-Based Cohort

Descriptive analyses were performed through continuous variables to measure central tendency and dispersion; medians were compared using the non-parametric Mann–Whitney *U*-test. Categorical variables such as demographic variables (sex and ethnicity) were compared using χ^2^ or the Fisher's exact test. Crude OR and 95% CI were assessed using an unconditional logistic regression to estimate the magnitude of risk associations between *GSTM1*/*GSTT1* deletions and *FLT3* mutations. Ethnicity was categorized into “blacks” and “non-blacks” as determined by the parents or guardians of the children, as a means of creating only two heterogeneity groups, since racial categorization in Brazil is a poor predictor of genomic ancestry (16). Age was also considered a categorical variable with three groups strata: (i) ≤ 2 years old; (ii) 2–10 years old; and (iii) >11 years old.

The OS was measured from the date of diagnosis to the last follow-up or death due to any cause. Patients who did not experience an event were censored at the time of the last known contact or at the date of the end of follow-up (December 31, 2017). The Kaplan–Meier survival analysis method was used to calculate the 5-year probabilities of OS, and estimated survival values were compared using the log-rank test to verify the association of one genetic alteration in the outcome of patients. An association between independent variables and the outcome was performed using the Cox proportional-hazard regression model with an estimated hazard ratio, and 95% CI were presented. Variables with *p* <0.25 in the univariate analysis (OS) or clinically relevant were included in the multivariate model following a stepwise forward method to adjust for confounders. All *p*-values were calculated using valid data and considered to be significant if <0.05. All analyses were performed using SPSS 20.0 (SPSS, Chicago, IL, USA, 2004).

## Conclusions

We speculate that the *GSTT1* polymorphism associated with prompt therapeutic protocols would permit survival improvements in the OS of patients with APL presenting a *GSTT1* null genotype.

## Data Availability Statement

The raw data supporting the conclusions of this article will be made available by the authors, without undue reservation, but following the General Data Protection Regulation (GDPR, current version OJL 127,23.5.2018, adapted in Brazil under name Lei Geral de Proteção de Dados Pessoais, LGPD 13.709).

## Ethics Statement

The studies involving human participants were reviewed and approved by The Ethical and Research Committee of the Instituto National de Câncer, Rio de Janeiro, Brazil (CEP#186.688). Written informed consent to participate in this study was provided by the participants' legal guardian/next of kin.

## Author Contributions

MSPO: conceptualization. FA, IS-C, GB, FS-B, DV, LM, ET-G, and EN: gene mutation tests. FA, SF, and LT: formal statistical analysis. MSPO and IR: resources. FA, SF, and MS: data curation. FA: writing—original draft preparation. MSPO, JW, and JC: writing—review and editing. MSPO and JW: supervision. MP and FA: project administration. All authors critically reviewed and approved the final draft of the manuscript.

## Conflict of Interest

The authors declare that the research was conducted in the absence of any commercial or financial relationships that could be construed as a potential conflict of interest.

## Brazilian Collaborative Study Group of Acute Leukemia (EMiLI)

Renata Sarkis Alves^1^, Renata Pereira de Souza Barros^1^, Rosania Maria Basegio^2^, Patrícia Carneiro de Brito^3^, José Carlos Córdoba^4^, Imaruí Costa^5^, Eloisa Cartaxo Eloy Fialho^6^, Teresa Cristina Cardoso Fonseca^7^, Isis Maria Quezado Magalhães^4^, Glaceanne Torres da Luz Mamede^6^, Eda Manzo^8^, Rebeca Ferreira Marques^9^, Gustavo Ribeiro Neves^10^, Andrea Gadelha Nobrega^6^, Claudia Teresa Oliveira^11^, Renato de Paula Guedes Oliveira^12^, Sidnei Epelman^12^, Ana Maria Marinho da Silva^13^, Luciana Nunes Silva^13^, Marcelo dos Santos Souza^2^, Regiana Quinto de Souza^7^, Adriano Nori Rodrigues Taniguchi^9^, Luciana Garcia Trujillo^14^, and Alayde Vieira Wanderley^15^.

^1^Hospital Estadual dos Servidores do Estado, RJ; ^2^Hospital Regional de Mato Grosso do Sul Rosa Pedrossian, Campo Grande, MS; ^3^Hospital Araújo Jorge, Goiânia, GO; ^4^Hematology-Oncology of Hospital da Criança de Brasília, DF; ^5^Hospital Infantil Joana de Gusmão, Florianópilis, SC; ^6^Hospital Napoleão Laureano, João pessoa, PB; ^7^Santa Casa, Hospital Manoel Novaes, Itabuna, BA; ^8^Hospital Amaral Carvalho, Jaú, SP; ^9^Hospital de Clínicas de Porto Alegre, Porto Alegre, RS; ^10^Gpaci Hospital Sarina Rolim Caracante, Sorocaba, SP; ^11^Hospital Estadual de Bauru, SP; ^12^Hospital Santa Marcelina, São Paulo, SP; ^13^Hospital Martagão Gesteira, Salvador, BA; ^14^Hospital Estadual Mario Covas, Santo André, SP; ^15^hospital Oncológico Infantil Octávio Lobo, Belém, PA.
