# Supplementary figures and images for: Pediatric Acute Promyelocytic Leukemia: Epidemiology, Molecular Features, and Importance of GST-Theta 1 in Chemotherapy Response and Outcome
Source: Front Oncol. 2021 Mar 19;11:642744. doi: 10.3389/fonc.2021.642744 (PMC8017304; doi:10.3389/fonc.2021.642744)

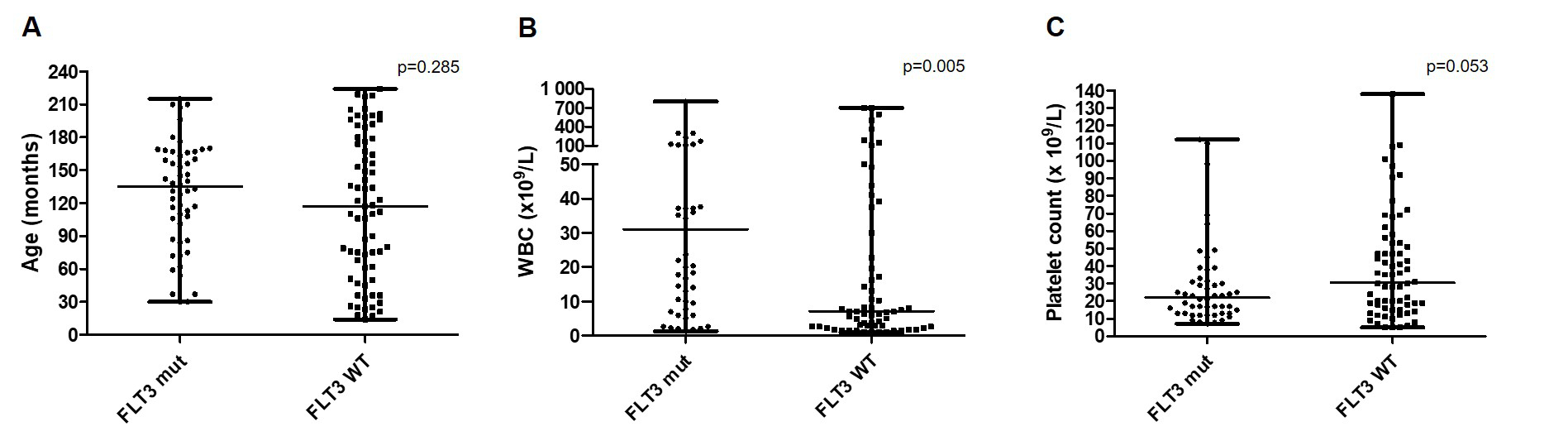

Supplement: Supplementary file 1 [file Image_1.JPEG]

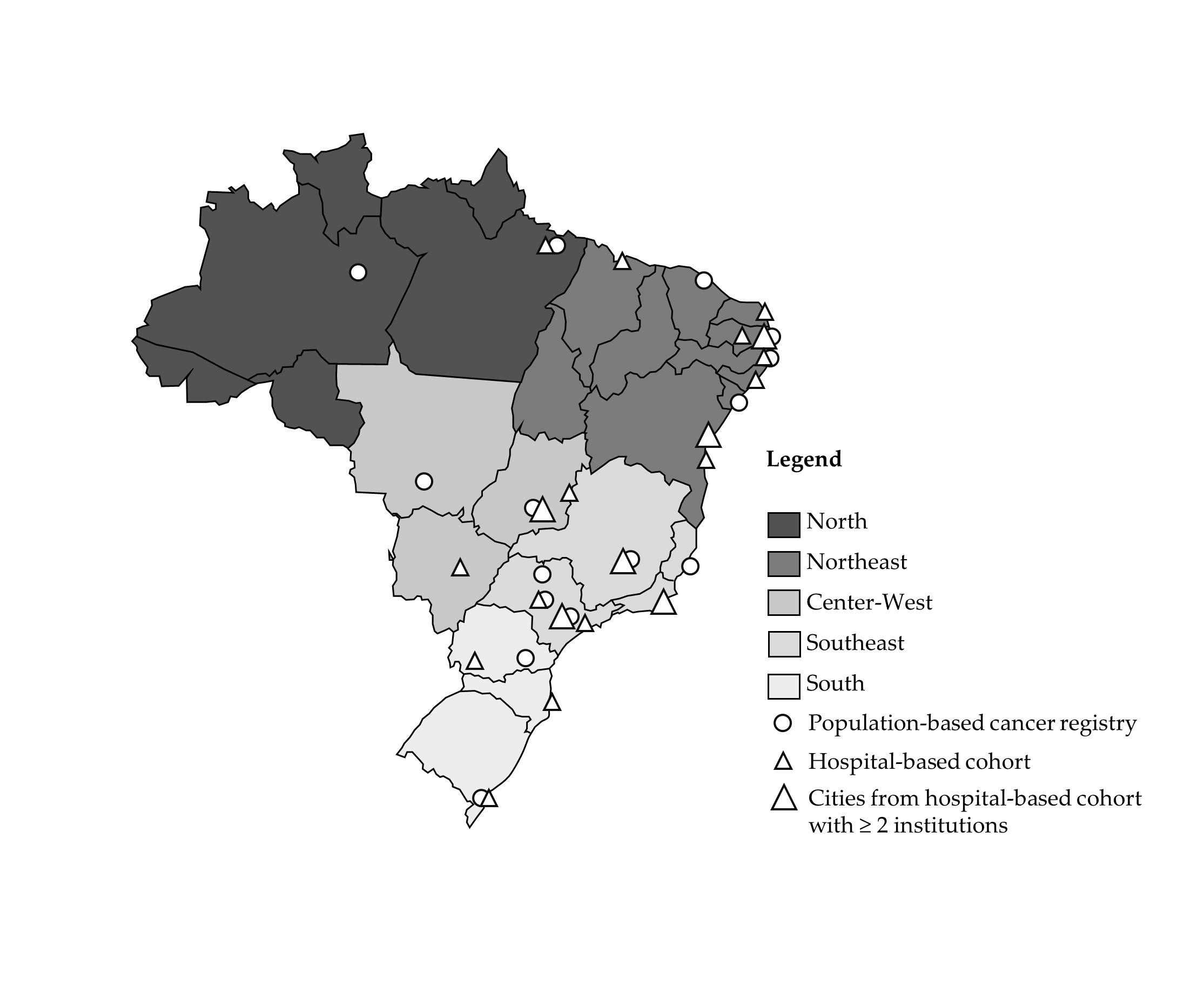

Supplement: Supplementary file 2 [file Image_2.JPEG]

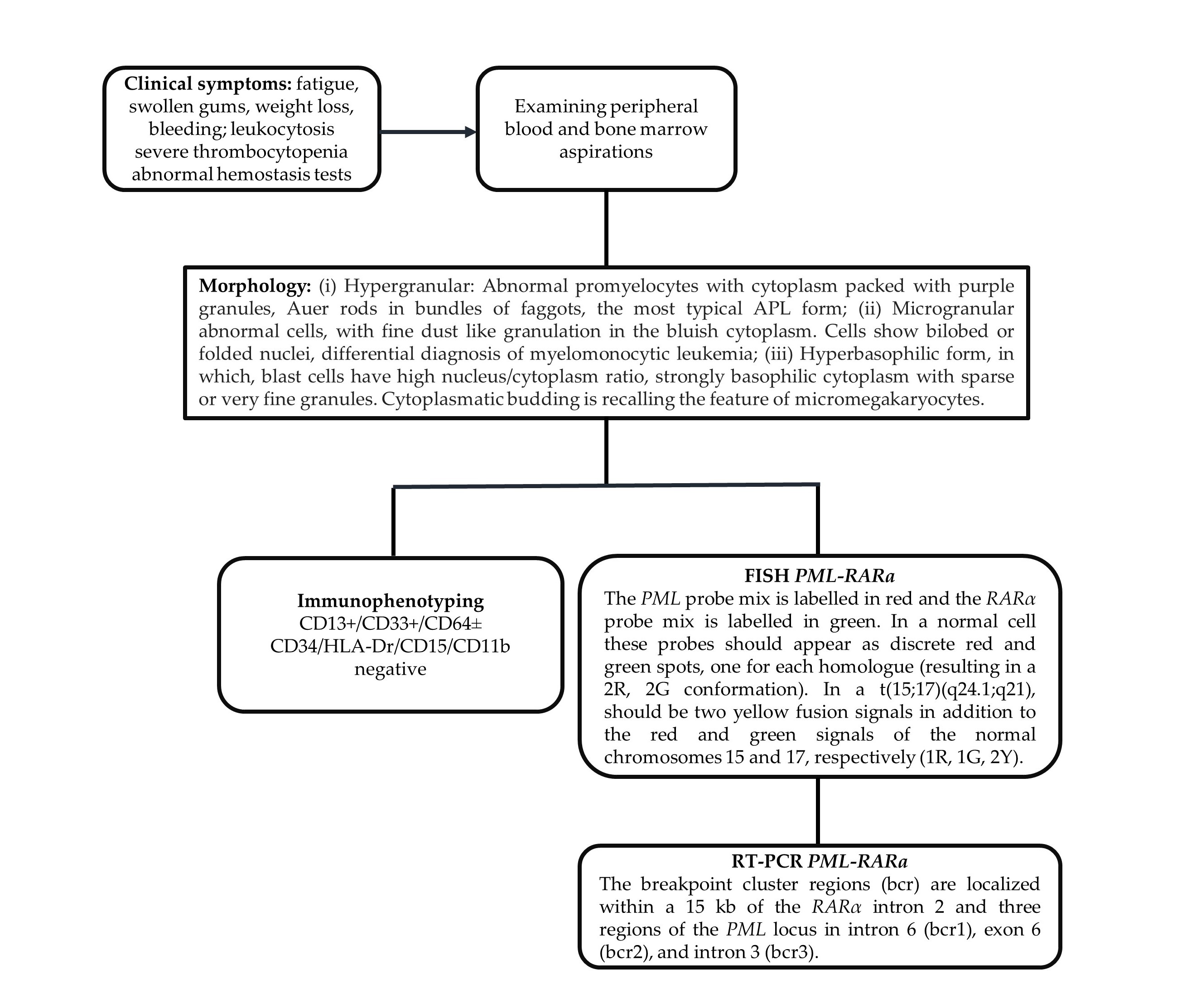

Supplement: Supplementary file 3 [file Image_3.JPEG]
