# Supplementary material for: Pediatric Acute Promyelocytic Leukemia: Epidemiology, Molecular Features, and Importance of GST-Theta 1 in Chemotherapy Response and Outcome
Source: Front Oncol. 2021 Mar 19;11:642744. doi: 10.3389/fonc.2021.642744 (PMC8017304; doi:10.3389/fonc.2021.642744)
Supplement: Supplementary file 4 [file Data_Sheet_1.docx]

Supplementary Material

# Supplementary Figures

**Supplementary Figure 1** Median comparison of age (A), WBC count (B), and platelet count (C) according to *FLT3* mutations.

**Supplementary Figure 2** Brazilian macro geographical regions. Dots in the Brazilian map represent the cities of the PBCR included triangles represent the institution locality from cases of the hospital-based cohort. Six cities present more than one institution.

**Supplementary Figure 3** Algorithm of tests for acute promyelocytic leukemia diagnosis.

# Supplementary Tables

**Supplementary Table 1** Crude incidence rates trend of pediatric Acute Promyelocytic Leukemia, 2000-2009

|  | **Calendar Years** | | | | | | | | | |
| --- | --- | --- | --- | --- | --- | --- | --- | --- | --- | --- |
|  | **2000** | **2001** | **2002** | **2003** | **2004** | **2005** | **2006** | **2007** | **2008** | **2009** |
| **North** | 0.00 | 0.00 | 0.00 | 0.00 | 0.00 | 0.00 | 0.00 | 0.07 | 0.15 | 0.00 |
| **Northeast** | 0.00 | 0.00 | 0.00 | 0.00 | 0.00 | 0.00 | 0.10 | 0.06 | 0.06 | 0.00 |
| **Center-West** | 0.00 | 0.00 | 0.00 | 0.00 | 0.00 | 0.00 | 0.00 | 0.29 | 0.00 | 0.00 |
| **Southeast** | 0.06 | 0.00 | 0.00 | 0.00 | 0.02 | 0.06 | 0.00 | 0.02 | 0.04 | 0.02 |
| **South** | 0.00 | 0.20 | 0.10 | 0.19 | 0.00 | 0.00 | 0.28 | 0.51 | 0.00 | 0.21 |
| **Brazil** | 0.03 | 0.02 | 0.01 | 0.02 | 0.01 | 0.03 | 0.05 | 0.10 | 0.05 | 0.03 |

**Supplementary Table 2** Allelic frequency of *FLT3* ITD mutations in pediatric acute promyelocytic leukemia

|  | **Allelic frequency ratio of *FLT3* ITD** | | |
| --- | --- | --- | --- |
|  | **≤0.35, n (%)** | **>0.35, n(%)** | ***p*** |
| **Age (years)** |  |  | **0.008** |
| ≤2 | - | - |  |
| >2-10 | 3 (21.4) | 10 (71.4) |  |
| ≥11 | 11 (78.6) | 4 (28.6) |  |
| **Sex** |  |  | **0.053** |
| Females | 8 (57.1) | 3 (21.4) |  |
| Males | 6 (42.9) | 11 (78.6) |  |
| **WBC count (x10^9^/L)** |  |  | **0.013** |
| ≤10 | 8 (57.1) | 1 (7.1) |  |
| >10 | 6 (42.9) | 13 (92.9) |  |
| **Platelet (×10^9^/L)** |  |  | **1.000** |
| ≤40 | 11 (78.6) | 10 (76.9) |  |
| >40 | 3 (21.4) | 3 (23.1) |  |

ITD, internal tandem duplication; WBC, white blood cell count. Statistical analysis performed with qui-square test.

**Supplementary Table 3** Risk associations between genetic polymorphisms and acute promyelocytic leukemia

| **Polymorphism** | **Controls (n = 397)**  **n (%)** | **APL (n = 115)**  **n (%)** | **Crude OR (95% CI)** | ***p*** |
| --- | --- | --- | --- | --- |
| ***GSTM****1* |  |  |  |  |
| Non-Null | 237 (59.6) | 66 (57.4) | Reference genotype |  |
| Null | 160 (40.3) | 49 (42.6) | 1.10 (0.72-1.68) | 0.658 |
| ***GSTT1*** |  |  |  |  |
| Non-Null | 307 (77.3) | 77 (67.0) | Reference genotype |  |
| Null | 90 (22.7) | 38 (33.0) | 1.68 (1.07-2.65) | 0.024 |

APL, acute promyelocytic leukemia; n, number of cases; OR, odds ratio.

**Supplementary Table 4** Univariate analysis of overall survival in pediatric APL, Brazil, 2002–2017

|  | **Univariate analysis*** | | |
| --- | --- | --- | --- |
|  | **n (events)**** | **5-year pOS, (SE)** | ***p*** |
| **Brazilian geographic region** |  |  | 0.915 |
| North/Northeast | 37 (9) | 66.5 (10.5) |  |
| Central-West | 27 (7) | 71.6 (9.3) |  |
| South/Southeast | 33 (10) | 64.6 (9.9) |  |
| **Age (years)** |  |  | 0.599 |
| ≤ 2 | 4 (1) | 66.7 (27.2) |  |
| > 2–10 | 46 (10) | 67.7 (9.6) |  |
| ≥ 11-19 | 47 (15) | 67.3 (7.0) |  |
| **Race** |  |  | 0.612 |
| Blacks | 6 (1) | 83.3 (15.2) |  |
| Non-Blacks | 84 (24) | 64.3 (6.7) |  |
| **WBC count (x10^9^/L)** |  |  | 0.625 |
| ≤ 10 | 55 (16) | 66.6 (7.4) |  |
| > 10 | 40 (10) | 67.0 (9.0) |  |
| **Platelet (×10^9^/l)** |  |  | 0.687 |
| ≤40 | 63 (16) | 67.1 (7.6) |  |
| >40 | 29 (8) | 63.8 (7.8) |  |
| **Morphologic subtype** |  |  | 0.964 |
| Hypergranular | 83 (22) | 66.6 (6.6) |  |
| Microgranular | 13 (4) | 69.2 (12.8) |  |
| ***PML-RARa*** |  |  | 0.646 |
| Positive | 77 (20) | 67.6 (6.8) |  |
| Negative | 12 (4) | 59.4 (16.0) |  |
| ***RAS* pathway mutations** |  |  |  |
| *FLT3* | 24 (8) | 57.9 (13.3) | 0.439 |
| *RAS* (mut vs. wt) | 9 (1) | 88.9 (10.5) | 0.234 |
| ***GSTT1* polymorphism** |  |  | 0.015 |
| Non-null | 52 (9) | 80.5 (6.1) |  |
| Null | 28 (11) | 48.2 (13.4) |  |
| ***GSTM1* polymorphism** |  |  | 0.476 |
| Non-null | 47 (13) | 66.2 (8.3) |  |
| Null | 33 (7) | 78.3 (7.3) |  |
| *Excluding early death. **Defined as death from any cause. CI, confidence interval; mut, mutated; pOS, probability of overall survival for 5 years; SE, standard error; wt, wild type. | | | |

**Supplementary Table 5** Cox regression model of the overall survival variables of acute promyelocytic leukemia, Brazil, 2002–2017

| **Variables** | **HR** | **95% CI** | ***p*** |
| --- | --- | --- | --- |
| Age (continuous) | 1.0 | 0.9-1.0 | 0.327 |
| WBC count (continuous) | 1.0 | 1.0-1.0 | 0.255 |
| Platelets (continuous) | 1.0 | 1.0-1.0 | 0.179 |
| *FLT3* mutations | 1.4 | 0.5-4.4 | 0.501 |
| GSTT1 null genotype* | 2.8 | 1.2-6.9 | 0.021 |

HR, hazard ratio; CI, confidence interval; WBC, white blood cells.

* Independent prognostic variable.

**Supplementary Table 6** Comparison of valid and missing data of cases investigates for *FLT3* mutations

|  | **Valid data*, n (%)** | **Missing data, n (%)** | ***p*** |
| --- | --- | --- | --- |
| **Age strata (years)** |  |  | 0.742 |
| ≤ 2 | 5 (4.2) | 3 (7.0) |  |
| > 2–10 | 52 (43.3) | 19 (44.2) |  |
| ≥ 11 | 63 (52.5) | 21 (48.8) |  |
| **Sex** |  |  | 0.040 |
| Females | 48 (40.0) | 25 (58.1) |  |
| Males | 72 (60.0) | 18 (41.9) |  |
| **Race** |  |  | 0.768 |
| Blacks | 11 (10.0) | 5 (12.2) |  |
| Non-Blacks | 99 (90.0) | 36 (87.8) |  |
| **WBC count at diagnosis (x10^9^/L)** |  |  | 0.513 |
| ≤ 10 | 53 (45.3) | 21 (51.2) |  |
| >10 | 64 (54.7) | 20 (48.8) |  |
| **Platelet (×10^9^/L)** |  |  | 0.967 |
| ≤40 | 79 (69.9) | 26 (70.3) |  |
| >40 | 34 (30.1) | 11 (29.7) |  |
| **Morphologic subtype** |  |  | 0.877 |
| Hypergranular | 104 (86.7) | 36 (85.7) |  |
| Microgranular | 16 (13.3) | 6 (14.3) |  |
| ***PML* breakpoint region** |  |  | 0.958 |
| Bcr 1 | 15 (34.9) | 3 (30.0) |  |
| Bcr 2 | 4 (9.3) | 1 (10.0) |  |
| Bcr 3 | 24 (55.8) | 6 (60.0) |  |
| ***RAS* mutations** |  |  | 0.098 |
| Mutated | 7 (7.0) | 4 (19.0) |  |
| Wild-type | 93 (93.0) | 17 (81.0) |  |
| ***GSTT1*** **polymorphism** |  |  | 0.846 |
| Non-null | 60 (67.4) | 17 (65.4) |  |
| Null | 29 (32.6) | 9 (34.6) |  |
| ***GSTM1*** **polymorphism** |  |  | 0.627 |
| Non-null | 50 (56.2) | 16 (61.5) |  |
| Null | 39 (43.8) | 10 (38.5) |  |
| **Early death*** |  |  | 0.208 |
| Yes | 38 (33.3) | 8 (22.2) |  |
| No | 76 (66.7) | 28 (77.8) |  |
| **Total** | 120 (73.6) | 43 (26.4) |  |

*Cases investigated for *FLT3* mutations according to biological material availability. **Death in the first 10 days after diagnosis. n, number of cases; WBC, white blood cell; wt, wild type. All p values were considered significant if less than 0.05

**Supplementary Table 7** Primers and polymerase chain reaction conditions for mutation detection

| **Gene *** | **Region** | **Amplicon size** | **Forward primer (5'–3')** | **Reverse primer (5'–3')** | **PCR conditions** |
| --- | --- | --- | --- | --- | --- |
| ***FLT3* D835** | Exon 20 | 114bp | CCGCCAGGAACGTGCTTG | CAGCCTCACATTGCCCC | 94°C 7’, 35 cycles of 94°C 30’’, 61°C 30’’, 72°C 45’’; 72°C 10’ ** |
| ***FLT3* ITD** | Exons 11/12 | 300bp | GCAATTTAGGTATGAAAGCCAGC | CTTTCAGCATTTTGACGGCAACC | 94°C 3’, 35 cycles of 94°C 30’’, 56°C 1’, 72°C 2’; 72°C 7’ |
| ***KRAS*** | Exon 1 | 220bp | AACCTTATGTGTGACATGTTC | ATGGTCCTGCACCAGTAAT | 95°C 30’’, 35 cycles of 94°C 30’’, 60°C 1’, 72°C 30’’; 72°C 10’ *** |
| ***NRAS*** | Exon 1 | 241bp | GACTGAGTACAAACTGGTGG | TGCATAACTGAATGTATACCC | 94°C 5’, 40 cycles of 94°C 1’, 57°C 1’, 72°C 1’; 72°C 10’ *** |
| ***PTPN11*** | Exon 3 | 384bo | CGACGTGGAAGATGAGATCTGA | CAGTCACAAGCCTTTGGAGTCAG | 94°C 8’, 33 cycles of 94°C 45’’, 58°C 30’’, 72°C 45’’; 72°C 10’ *** |
| * Reference sequences: *FLT3* NG_007066.1; *KRAS* NG_007524.1; *NRAS* NG_007572.1; *KIT* NG_007456.1; *PTPN11* NG_007459.1. **PCR followed by restriction fragment length polymorphism with EcoRV enzyme (Biolabs, New England, UK) for 37°C, overnight, following fabricant recommendations. ***PCR followed by Sanger sequencing. Bp, base pairs; PCR, polymerase chain reaction. | | | | | |
